# The Role of Phytochemicals in Aging and Aging-Related Diseases

**DOI:** 10.3390/ph18111713

**Published:** 2025-11-11

**Authors:** Alessandro Medoro, Giovanni Scapagnini, Sergio Davinelli

**Affiliations:** Department of Medicine and Health Sciences “V. Tiberio”, University of Molise, 86100 Campobasso, Italy

Over the past century, advances in medical care, hygiene, and nutrition have greatly extended human lifespan. However, this demographic achievement has not been accompanied by a corresponding extension of healthspan, the period during which individuals remain functionally independent and largely free from chronic disease. One of the major goals of the biology of aging is to preserve health throughout life rather than merely extend survival, by counteracting the molecular processes that compromise cellular integrity and enhancing adaptive resilience [1]. Among the determinants of healthy aging, plant-based diets rich in phytochemicals may influence longevity and preserve health during aging [2].

Therefore, this Special Issue entitled “The Role of Phytochemicals in Aging and Aging-Related Diseases” was framed around a simple question: can phytochemicals modulate the biology of aging and influence the trajectory of age-related diseases? The eleven original articles and reviews collected here provide a consistent perspective through diverse methods related to this topic. Beyond their canonical antioxidant capacity, plant-derived molecules can modulate key biological processes underlying aging, including chronic low-grade inflammation, cellular senescence, mitochondrial dysfunction, and lipid metabolism. Taken together, these contributions show how phytochemicals may influence a number of biological processes that support healthy aging.

The initial article focuses on the vascular field. An elegant study in apolipoprotein E knockout (*ApoE*^−^/^−^) mice by Yigit et al. showed that white tea infusion, rich in polyphenols, improved plasma lipid profile, reduced vascular inflammation and oxidative stress, and decreased lesion burden. This in vivo evidence indicates how a complex matrix of phytochemicals may reshape a multifactorial atherosclerotic phenotype [3]. This vascular protection is consistent with computational investigations from our group on algal carotenoids as a potential inhibitor of proprotein convertase subtilisin/kexin type 9 (PCSK9), a protease that drives the degradation of hepatic low-density lipoprotein (LDL) receptors and raises circulating LDL cholesterol. Astaxanthin emerged as the top-scoring ligand, suggesting that this xanthophyll could preserve LDL receptor expression and improve cholesterol homeostasis. Rather than replacing current biological PCSK9 inhibitors, these findings may support the rationale of nutraceutical supplementation, particularly in individuals with moderate cardiovascular risk or in secondary prevention settings where adherence to pharmacotherapy may be suboptimal. Consistent with this computational evidence, clinical and preclinical studies indicate that astaxanthin improves lipid profile, enhances insulin sensitivity, and activates cytoprotective signaling through phosphatidylinositol 3-kinase (PI3K)–AKT and nuclear factor erythroid 2-related factor 2 (Nrf2), while at the same time attenuating nuclear factor κB (NF-κB) and systemic inflammation [4,5]. The role of Nrf2 is further examined in a review by Gjorgieva Ackova et al. on alkaloids as inhibitors of this pathway, where Nrf2 emerges as a context-dependent target: transient activation protects cells from oxidative injury, whereas constitutive overactivation in cancer cells can sustain survival and chemoresistance. Selective inhibition with alkaloids may restore chemosensitivity or exploit cancer redox vulnerabilities, exemplifying the context-dependent nature of Nrf2 as a therapeutic target [6].

The potential therapeutic application of major classes of dietary phytochemicals in the nervous system is discussed in a review by Dareowolabi et al., which highlights how these molecules affect the interplay of oxidative stress, neuroinflammation, and mitochondrial dysfunction in age-related neurodegenerative disorders. In particular, the authors emphasize the role of these molecules in modulating oxidative stress, controlling neuroinflammation, and supporting mitochondrial function through the regulation of Nrf2 signaling, the NF-κB inflammatory cascade, and the PTEN-induced kinase 1 (PINK1)–Parkin pathway in neurodegenerative disorders. Interestingly, they point out that absorption, bioavailability, blood–brain barrier permeability, and microbiota-driven metabolism critically influence neuroprotective efficacy [7]. Another experimental study demonstrated that physcion, an anthraquinone found in many medicinal plants and vegetables, attenuates microglial activation and pro-inflammatory cytokine release in a lipopolysaccharide (LPS)-induced model of neuroinflammation, upregulating Nrf2 and heme-oxygenase-1 (HO-1) and restoring synaptic proteins. These findings are particularly relevant to “neuro-inflammaging”, a state of chronic, low-grade brain inflammation that accelerates cognitive decline. Together, these studies define phytochemicals as modulators of neural stress-response networks, acting beyond generic anti-inflammatory mechanisms [8].

A persistent, dysregulated activation of the immune system may contribute to the chronic low-grade inflammation that characterizes aging and age-related diseases. In infectious conditions such as periodontitis, resident cells of the gingival tissue can sustain and perpetuate the inflammatory response, a process that tends to worsen with age as immune regulation declines. Gingival fibroblasts, a major source of inflammatory cytokines released during periodontal infection, therefore represent a useful experimental model to study these mechanisms. In this context, Kurek-Górecka et al. investigated the effects of apigenin and quercetin, two flavonoids abundant in propolis, on human gingival fibroblasts stimulated with lipopolysaccharide (LPS) and interferon-α. The authors observed a modulation of cytokine secretion, particularly IL-6 and IL-15, suggesting that polyphenols may mitigate infection-driven inflammation and contribute to the maintenance of immune homeostasis during aging [9].

An additional study conducted by Jang et al. investigated a standardized water extract containing *Angelica gigas* roots and *Pueraria lobata* flowers, which enhanced immune function in macrophages and immunosuppressed mice through the activation of Toll-like receptor 2 and 6 (TLR2/6), which are key receptors involved in microbial sensing and inflammatory signaling via the NF-κB and MAPK pathways. Beyond restoring cytokine production and natural killer (NK) cell activity, the extract preserved intestinal barrier integrity, modulated gut microbial diversity, and re-established correlations between commensal bacteria and TLR2/6 signaling. These findings highlight the emerging concept of a gut–immune–brain axis, where the fine-tuning of innate receptors and epithelial interfaces contributes to systemic immune resilience during aging [10].

Given the reciprocal interplay between inflammation and aging, this Special Issue also addresses the theme of cellular senescence, which is closely linked to the secretion of inflammatory mediators. Senescence represents a cellular stress response triggered by DNA damage, telomere attrition, and mitochondrial dysfunction, among other stimuli, leading to the stable arrest of the cell cycle. Senescent cells lose their physiological function, become resistant to apoptosis, and secrete a pro-inflammatory senescence-associated secretory phenotype (SASP), which contributes to tissue degeneration and chronic inflammation. Persistent inflammatory signals, in turn, promote cellular senescence, creating a self-sustaining loop that links oxidative stress, mitochondrial impairment, and inflammaging [11]. In this context, senolytic phytochemicals, compounds capable of selectively eliminating senescent cells, represent a promising area of development in anti-aging research. In human dermal fibroblasts, Park et al. demonstrated that ε-viniferin, a resveratrol dimer, reduces mitochondrial oxidative stress and rejuvenates senescent cells through regulation of the *RGS16* gene. The compound decreased mitochondrial ROS production, improved bioenergetic efficiency, and activated mitophagy, promoting the clearance of damaged mitochondria. As a selective senolytic, ε-viniferin eliminated senescent fibroblasts without affecting proliferating cells and reduced senescence markers. These effects were mediated by *RGS16* upregulation, reproducing the antioxidant and mitochondrial-restorative actions of the compound [12].

Two contributions shed light on the musculoskeletal axis of healthy aging. Shaikh et al. present a conceptual framework linking age-related physiological decline to chronic low-grade inflammation and mitochondrial dysfunction. They emphasize that the loss of metabolic and neuromuscular integrity underlying sarcopenia and frailty requires integrated strategies that combine lifestyle modification, regular exercise, and targeted nutraceutical interventions. Among these, phytochemicals with mitochondrial-modulating properties, also involved in myogenesis and muscle regeneration, are promising candidates for preserving muscle mass and functionality, and for preventing or slowing down the progression of physical frailty [13]. Hong et al. investigated the osteogenic potential of an *n*-hexane extract from *Cotoneaster microphyllus* Nakai, an endemic species of the Korean Peninsula. In MC3T3-E1 pre-osteoblast cells and ovariectomized mice, the extract increased the signaling activation of osteoblast differentiation and the expression of osteogenic markers. These coordinated actions stimulated bone formation while reducing bone resorption, suggesting anti-osteoporotic potential. Osteoporosis, a major cause of morbidity in aging populations, reflects a progressive imbalance between bone formation and degradation that parallels the loss of muscle mass and strength [14]. Overall, these findings support the concept of a “bone–muscle unit” approach based on phytochemicals as a means to preserve mobility and independence in aging populations.

The closing contribution of this Special Issue integrates computational and experimental strategies to accelerate the discovery of compounds with potential anti-aging properties. Combining ligand- and structure-based virtual screening of resveratrol-like polyphenols with validation in zebrafish models, Hernández-Silva et al. identified molecules capable of modulating key processes of aging, including telomerase activity and inflammation. Resveratrol and sakuranetin improved telomerase-related parameters, whereas apigenin, genistein, and hesperetin exerted significant anti-inflammatory effects. The integration of in silico prediction with in vivo experimentation provides a rigorous and efficient framework for identifying bioactive molecules targeting fundamental cellular aging mechanisms [15].

Across all contributions, three cross-cutting challenges emerge. First, the profile of absorption and tissue distribution remains a critical limitation for many phytochemicals, highlighting the need for innovative delivery systems and a better understanding of microbiota-mediated metabolism. Second, the non-linear dose–response relationships typical of hormesis must be carefully addressed, as highlighted by the divergent effects of phytochemicals in the Nrf2 activation and inhibition. Third, clinical translation remains an unmet need, requiring rigorously designed trials with functional and patient-centered endpoints to determine whether the promising preclinical findings can be translated into measurable improvements in healthspan.
